# Size Matters: Molecular Weight Specificity of Hyaluronan Effects in Cell Biology

**DOI:** 10.1155/2015/563818

**Published:** 2015-09-10

**Authors:** Jaime M. Cyphert, Carol S. Trempus, Stavros Garantziotis

**Affiliations:** Laboratory of Respiratory Biology, Department of Intramural Research, National Institute of Environmental Health Sciences, Research Triangle Park, NC 27709, USA

## Abstract

Hyaluronan signaling properties are unique among other biologically active molecules, that they are apparently not influenced by postsynthetic molecular modification, but by hyaluronan fragment size. This review summarizes the current knowledge about the generation of hyaluronan fragments of different size and size-dependent differences in hyaluronan signaling as well as their downstream biological effects.

## 1. Hyaluronan: A Simple Sugar with Multifaceted Biological Effects

If there were ever a competition for the most simply designed, yet most versatile biological molecule in nature, hyaluronan (HA) would be a strong contender. Built of simple disaccharide sequences (D-glucuronic acid and D-N-acetylglucosamine, bound through alternating *β*-1,4 and *β*-1,3 glycosidic bonds), and with no known postsynthetic modification, HA was long mistaken for an inert filler of the extracellular space. In the past decades, however, it has become evident that HA possesses manifold signaling properties. A fascinating, if perhaps vexing, insight has also been that HA will often demonstrate opposing actions: it can have pro- or anti-inflammatory properties, promote or inhibit cell migration, activate, or stop cell division and differentiation. It appears that three main factors dictate the effect of HA: one is cell-specific (receptor expression, signaling pathways, and cell cycle); two are HA-related (size and location). This review will focus on HA sizes and their effect on HA signaling and biological effects.

Size dependency of signaling raises interesting questions. Why would a larger or smaller molecule of identical, monotonous molecular structure have different effects? Presumably HA receptors need a minimal molecular size to engage the ligand, but further size increase should have no effect on receptor recognition. Two concepts which help explain this observation have gathered experimental support. The first concept suggests that HA size may influence affinity to receptors; also, receptor complexes may cluster differently depending on HA size. The second concept, less well understood, is that size may affect HA uptake by the cell, and HA intracellular signaling may also modulate biological responses.

An additional impediment on the elucidation of size-dependent HA signaling and biological effects is the confusing language that is used in scientific publications. While everyone seems to agree on designating HA over 1 million Da “high molecular weight,” the nomenclature of smaller-size HA is nebulous. Different papers use interchangeably terms such as “short-fragment HA,” “low molecular weight HA,” and “HA oligosaccharides” to describe HA molecules from a few disaccharides up to over 700 kDa. For this review we are using the following nomenclature, for expediency's sake: HA of approximately 20 monosaccharide length or less (the minimum that differentiates between monovalent and divalent interactions with CD44 [1]) are “HA oligosaccharides (oHA)”; HA of over 1 million Da (the minimum that is thought to be native HA [2]) is “high molecular weight (HMW-HA)”; everything in between is “low molecular weight (LMW-HA)”. For scientific papers it may be better to simply define the size or range of sizes the investigators are working with, until a clear mechanistic understanding of fragment size classification emerges.

In the following we will provide brief overviews of the mechanisms of HA synthesis and degradation, which lead to the generation of different fragment sizes; the current state of knowledge on HA size-dependent signaling; and a conclusive discussion of implications and future directions.

## 2. Hyaluronan Synthesis

HA is uniquely synthesized at the plasma membrane rather than in the Golgi apparatus as is typical of other glycosaminoglycans (GAGs) [3]. Synthesis of mammalian HA is accomplished by a family of membrane-bound glycosyltransferases composed of three isozymes, hyaluronan synthases (HAS) 1, 2, and 3. HAS enzymes are evolutionarily conserved and are highly homologous (55–70% protein identity) [4, 5], and they catalyze the addition of UDP-D-glucuronic acid (GlcA) and UDP-N-acetyl-D-glucosamine (GlcNAC) monomers in an alternating assembly to form HA polymers [6, 7]. Although the three HAS isoforms are similar and synthesize an identical product, they exhibit differences in half-life and stability, the rate of HA synthesis, and affinity for HA substrates, all of which potentially affect the regulation of HA synthesis and biological function [8]. Of particular interest is the finding that the three HAS enzymes synthesized HA of varying molecular masses. In general, HAS3 synthesized the shortest HA polymer sizes (1 × 10^5^ to 1 × 10^6^ Da), while HAS1 and HAS2 synthesized larger polymers (2 × 10^5^ to 2 × 10^6^ Da), although the major population of HA produced by HAS2 tended to be concentrated on the longer end of the spectrum (>2 × 10^6^ Da) compared to HAS1, which generated a wider range of HA polymers [8]. Because of the biological differences exhibited by HA of differing polymer lengths, the innate biochemical and synthetic capabilities of the HAS enzymes may serve an important regulatory role in development, injury, and disease.

The HAS genes exhibit different temporal patterns of expression during morphogenesis [4]. HAS2 is expressed throughout all stages of embryogenesis [9] and is considered to be the major hyaluronan synthase during development. Camenisch et al. determined that, of the three HAS isoenzymes, only HAS2 was indispensable, with embryonic lethality due to severe cardiac and vascular defects at midgestation in HAS2^−/−^ mice [10]. HAS1 and HAS3 expressions, on the other hand, are restricted to early and late stages of development, respectively, although expression overlaps with HAS2 [9]. HAS1 and 3 deficient mice as well as HAS1 and 3 double knockouts are both viable and fertile [9].

At the tissue level, HAS gene expression and subsequent HA synthesis is regulated by a wide range of cytokines and growth factors (reviewed in [2, 11]). All three HAS genes may respond similarly to a particular signal, or there may be a differential response, as demonstrated by TGF-*β*1-mediated downregulation of HAS3 but upregulation of HAS1 expression, seen in a dose-dependent manner in human fibroblast-like synoviocytes [12]. Dysregulation of HAS gene expression plays important roles in disease and injury, consistent with the biological roles of HA in disease progression, wound healing, and tissue regeneration. In cancer, overexpression of hyaluronan synthases influences tumor growth, metastatic potential, and progression in several malignancies, including prostate, colon, breast, and endometrial cancers (reviewed in [13]). Ectopic expression of HAS genes may also functionally alter the biological responses of cells to injury* in vivo* [14, 15] (reviewed more extensively below). Taken together, available studies suggest that HA synthases are critical mediators in development, injury, and disease.

## 3. Degradation of Hyaluronan by Hyaluronidases

The mechanism of HA removal or turnover is facilitated in part by hyaluronidases (HYALs), which, in mammals, consists of a family of enzymes including hyaluronidases 1–4 (HYAL1–4), PH20, and HYALP1 [16, 17]. HYALs are also found in lower organisms, like bacteria, which catabolize HA to generate primarily disaccharides and in part facilitate mobility within tissue [2], and in leeches and crustaceans, which produce predominately tetra- and hexasaccharide fragments [18]. HYALs as a class are highly homologous endoglycosidases, and in terms of HA catabolism, they specifically hydrolyze the *β*-1,4 linkage of the HA molecule, which is a linear polysaccharide composed of repeating *β*-1,4-linked D-glucuronic acid (GlcA) and *β*-1,3-linked* N*-acetyl-D-glucosamine (GlcNAc) disaccharide units [17–19]. The range of activity of mammalian HYALs is not strictly limited to HA as they can also degrade chondroitin sulfates, although prokaryotic HYALs specifically act on HA [17, 20]. HYALs can be further broken down into distinct groups; for example, HYAL1–HYAL3 are primarily active at an acidic pH, while PH20 has optimum activity at a neutral pH [16, 21].

Of the 6 HYAL family members, HYAL1 and HYAL2 are the predominant isoforms functioning to catabolize HA in somatic tissues. Triggs-Raine et al. [22] detailed a broad mRNA expression pattern for HYAL2 (heart, skeletal muscle, colon, spleen, kidney, liver, placenta, and lung), while HYAL1 is more limited in scope but is high in liver (which is a primary location of HA degradation) and is less strongly expressed in heart, spleen, kidney, and lung; the HYAL1 protein is also found in plasma and urine. HYAL3 is even more limited in its expression pattern, with low levels in brain, liver, testis, and bone marrow [22]. PH20/SPAM1 is specific to sperm and has a role in fertilization [16]. Finally, HYAL4 is a chondroitinase with no evidence of HA catabolic activity [23], with expression in the placenta and skeletal muscle [16], and HYALP1 is an expressed pseudogene [16, 17].

HA degradation into smaller fragments is accomplished by HYAL1 and HYAL2 acting in concert to catabolize HA into tetrasaccharides. HMW-HA is anchored to the cell surface through CD44 and HYAL2 and localized to lipid rafts in the cell membrane. The acidic environment necessary for HYAL2 activity is provided by NHE2 [24], facilitating the generation of HA polymers of approximately 20 kDa (or 50 disaccharide units). In this model, 20 kDa fragments are internalized and transported first to endosome and then to lysosomes, where lysosomal HYAL1 further degrades the HA into tetrasaccharide units (reviewed in [20]). Experimental details and confirmation of this model are still outstanding. The significance of HYAL-mediated degradation of HA is demonstrated in mucopolysaccharide hyaluronidase deficiency, first described by [25]. This disorder is characterized by elevated HA levels in the plasma (>38–90-fold increase over normal plasma levels) concomitant with lack of hyaluronidase activity [25]. This lysosomal storage disorder is now termed mucopolysaccharidosis IX [22], and subsequent characterization revealed that hyaluronidase activity is specifically abrogated through mutations in HYAL1. The disease has a relatively mild phenotype, limited to specific cell types (fibroblasts and histiocytes) and characterized by accumulation of HA, short stature, and multiple soft tissue masses in the joints. A further demonstration of how HYALs contribute to developmental processes was shown with a mouse model of HYAL2 deficiency, which, similar to mucopolysaccharidosis IX in humans, was characterized by increased plasma concentrations of HA, and a relatively mild phenotype, in this case with mild craniofacial and hematological defects [26]. Interactions with other genetic loci are suspected, as HYAL2 deficiency in an outbred mouse resulted in much more severe cardiopulmonary pathology and early mortality compared to HYAL2 deficiency in an inbred genetic background [27]. Increased HYAL levels have also been found in several carcinomas, including prostate and bladder, as well as breast and head and neck cancer, and tend to correlate with more invasive and metastatic phenotypes (reviewed in [21]). Cumulatively, this suggests that HYALs have distinctive roles in developmental and disease processes through the regulation of HA metabolism.

Although many diverse biological responses have been attributed to HA and its various size polymers, interpretation of experimental findings, both* in vivo* and* in vitro*, may be complicated by, for example, low levels of bacterial contamination, which may independently activate key HA receptors. Recently, Muto et al. [28] developed mouse lines overexpressing HYAL1, in an attempt to generate HA fragments* in vivo* independently of other mitigating factors. Using models of constitutive overexpression of HYAL1 in mouse skin (K14/HYAL1) and tamoxifen-inducible expression localized to the epidermis (K14CreERT/HYAL1), they determined that HA catabolism in the absence of injury* in vivo* initiated dendritic cell (DC) migration and maturation, which in turn muted the response to contact hypersensitization (CHS). Interestingly, HYAL1-mediated catabolism of HMW-HA into size ranging between 0.5 and 27 kDa (tetrasaccharides to 68 mer disaccharides) did not result in any phenotypic or inflammatory changes, so in the absence of any specific injury or challenge, catabolism of HA to smaller polymers by HYAL1 did not alone induce an immune response in these models. These results were recapitulated by injection of tetrasaccharide oHA into the skin of wild type mice, resulting in increased migration of DCs out of the skin and functionally, a diminished CHS response. Finally, they also showed that, in this context, HYAL1 or oHA function is dependent upon TLR4. While HYAL1 overexpression or oHA injection in a TLR4^−/−^ background resulted in HA fragmentation, there was no change in cutaneous DC levels in either case. This effect was not seen with HYAL1 on a CD44 deficient background, demonstrating specificity for TLR4 in this process [28]. HYAL1 is only active at a low pH, which is unlikely to have been present in the uninflamed skin of these mice. Therefore, the exact mechanism of HYAL1 activity in this case and in inflammation generally is still far from completely understood. However, it should be noted that much is unknown about hyaluronidase activity and function* in vivo*, and it is possible that posttranslational processing or other factors (association with proteins or salts) substantially change activity and specificity from what is found* in vitro* [29, 30].

## 4. Degradation of Hyaluronan by Nonspecific Pathways

Aside from the specific enzymatic degradation pathways described above, HMW-HA can be fragmented by nonspecific pathways as well. Reactive oxygen species (ROS), including superoxide, hydrogen peroxide, nitric oxide and peroxynitrite, and hypohalous acids (reviewed in [31]), are generated during the inflammatory response in sepsis, tissue inflammation, and ischemia-reperfusion injury and can degrade HA [31]. The most direct evidence for this has been accumulated in the synovial fluid, where inflammatory oxidation leads to degradation of native HMW-HA with resulting decrease in synovial fluid viscosity and cartilage degeneration, and in the airways, where ROS can degrade luminal epithelial HA [32]. It should also be mentioned that nonenzymatically produced, ambient ROS can also generate HMW-HA breakdown products. This may be most relevant in environmental lung injury; for example, inhaled ozone and chlorine gas can generate LMW-HA from HMW-HA* in vitro*. No matter the origin, it seems clear that excessive generation of ROS contributes to a proinflammatory status by the oxidative degradation of hyaluronan. The corollary to this is that neutralization of ROS, for example, through superoxide dismutase, decreases HMW-HA degradation and inflammation [33–35]. Finally, the possibility may be entertained that ROS scavenging is in fact one of the physiological functions of HMW-HA as was proposed recently [36]; however, no experimental support for this hypothesis exists at this point. Beyond this hypothesis, it should also be noted that degradation of HMW-HA by ROS may also have salutary effects, such as the promotion of ciliary beating in the airways [37]. Thus, ROS may engage hyaluronan in a fine-tuned interaction rather than a monolithic response, and in fact hyaluronan may be involved in the emerging signaling pathway for these molecules [32, 38].

## 5. Effects of Hyaluronan Size on Cell Receptor Signaling

As part of the extracellular matrix (ECM), HA plays an important role in the maintenance of appropriate cell-cell communication. When ECM homeostasis is disrupted during pathological conditions (tumor invasion, inflammation, tissue remodeling, etc.) endogenous HMW-HA can be degraded by hyaluronidases [39] and reactive oxygen species [31] into LMW-HA, which can be further depolymerized to oHA. HA and its degradation products bind several cell surface receptors such as CD44, RHAMM, HARE, LYVE1, layilin, TLR2, and TLR4 [15, 40–49], where the size of HA can have a significant influence on receptor activation and its downstream signaling. One theory proposes that signal transduction by HA is dependent on multivalent and cooperative interactions and/or the ability of HA to cluster the receptors on the membrane [15, 50]. For example the interaction of CD44 and HA is strongly influenced by cell-specific factors, cell type, state of activation, and HA size. Different sizes of HA have distinct effects on CD44 clustering: HMW-HA increases clustering strength while oHA has no apparent effect. However, sequential addition of oHA following HMW-HA reduces the clustering strength induced by HMW-HA [51]. Long chains of HA possess multivalent sites for CD44 binding while oHA have only 1 or 2 binding sites [1, 52] suggesting that oHA binding can act as an antagonist by replacing these interactions with low affinity, low valency interactions [50].

HA of different sizes can also signal through toll-like receptors (TLRs), either independently or in concert with other HA receptors. LMW-HA has been shown to engage in a unique receptor complex of CD44 and TLRs to induce cytokine release [53] and airway hyperresponsiveness (AHR) [54, 55] in macrophages and naïve mice, respectively. However, in a model of bleomycin-induced acute lung injury LMW-HA required both TLR2 and TLR4, along with MyD88, to stimulate chemokine expression, which was independent of CD44. HMW-HA was protective in this model and also required TLR2, TLR4, and MyD88 [15]. Short oHA have also been shown to transmit various “danger signals” and to signal through TLRs [56]. Interestingly, receptor binding and activation by oHA can even differ depending on the number of disaccharides present. For example, 4-mer oHA interacts with TLR2 and TLR4, but not CD44, while 6-mer oHA can bind to TLR2, TLR4, and CD44 [41, 57–59]. Additionally, 6- to 18-mer oHA bind monovalently to CD44, whereas larger polymers bind multivalently [60], which can affect clustering and signaling of this receptor. Furthermore, <6-mer oHA have low affinity for TSG-6, which is required for HA transfer to the inter-alpha-trypsin inhibitor (I*α*I) heavy chain and optimal signaling [61]. Conversely, 8- to 21-mer oHA induce an irreversible transfer of TSG-6 to the HA moiety and thus can compete with HA signaling by removing TSG-6 from the binding pool [62].

Size-dependent HA signaling can also differ according to cell type. LMW-HA induces activation and maturation of dendritic cells via TLR4, independently of RHAMM, CD44, and TLR2, to induce phosphorylation of p38/p42/44 MAPK and NF-*κ*B [41]. Conversely, LMW-HA stimulates macrophages independently of CD44 and TLR4 via the TLR2/MyD88 pathway, and HMW-HA can act as a competitive inhibitor of this response [63]. Oligosaccharide HA can activate TLR4 and CD44 pathways in chondrocytes [57, 64, 65], while in synovial fibroblasts it activates TLR2 and TLR4, but not CD44 [66], and in vascular endothelial cells it activates RHAMM [67]. In aggregate, available evidence suggests that HA size influences receptor complex formation in a size-specific manner and thus modifies downstream signaling cascades.

## 6. Intracellular HA Signaling

HA is normally produced by synthases which reside at the cell membrane and is immediately extruded to the extracellular space without need for shuttling or exocytosis. Thus, most HA resides extracellularly and exerts its function in that space. However, HA has also been detected intracellularly, where it associates with the mitotic spindle, microtubules, and the receptor RHAMM [68, 69]. Two possible mechanisms by which HA may be localized intracellularly and potentially to contribute to signaling activity have been identified: intracellular (physiological or aberrant) production of HA and uptake of extracellular HA.

HA synthesis can deviate from the normal pattern, especially in malignancy and cell injury. Various hematologic malignancies such as monoclonal gammopathy of undetermined significance, multiple myeloma and Waldenström's macroglobulinemia as well as solid cancers such as bladder cancer feature cells with aberrant splice variants of HAS1, which are associated with cancer diagnosis, relapse and poor outcome (reviewed in [70]). Accumulating evidence has led to the hypothesis that HAS1 in these cancer cells may produce intracellular HA which competes with the mitotic apparatus for RHAMM binding and thus protect the cells from RHAMM-mediated mitotic arrest [70]. In conditions of endoplasmic reticulum (ER) stress, injured cells also produce HA cables that appear to emanate from a perinuclear region and protrude through the cytoplasm into the extracellular space [71]. This intracellular HA directly communicates with the extracellular space and allows inflammatory cells to congregate into the inflammatory site [71].

Uptake of extracellular HA seems to be directly tied to its digestion. HA of higher molecular weight can be digested by a variety of enzymatic and nonenzymatic [18, 29, 32, 72, 73] pathways to 50–100 long saccharide polymers and then be taken up by the cell either through receptor binding (CD44, RHAMM, LYVE-1, HARE) [74, 75] or through pinocytosis [76]. Much (or most) of the endocytosed HA is localized to the endosome and lysosome [68], where it is digested to oligosaccharides by hyaluronidases 1 and 2, and probably further by *β*-D-glucuronidase and *β*-N-acetyl-D-hexosaminidase [77]. The activities of at least HYAL1 and the extracellular *β*-hexosaminidase appear to be partly redundant [78]. However the regulation of HA catabolism and the fate of these fragments are unclear. They could be recycled for the generation of glycosaminoglycans, they could be exocytosed and engage extracellular HA receptors, or they could also engage intracellular HA receptors. The presence of HA receptors (notably RHAMM) in the cytoplasm strongly suggests that intracellular HA can have signaling activity. Evanko et al. have shown that intracellular HA, RHAMM, and microtubules colocalize in the cytoplasm and the nucleus [68] and may affect the mitotic apparatus and directly or indirectly modulate RHAMM-mediated signaling [68].

## 7. Size Dependence of HA Biological Effects

Although the specific mechanisms involved in the diverse signaling of HA are still poorly understood, it is known that HA can modulate many biological effects including cell adhesion, cell migration, morphogenesis, tumorigenesis, cell survival, apoptosis, and inflammation and that these biological effects can differ depending on HA size. Endogenous HMW-HA has been shown to be anti-inflammatory and antiangiogenic [11]. Interestingly, depending on cellular localization, endogenous HMW-HA can either protect from epithelial apoptosis in lung injury [15] or promote an invasive fibroblast phenotype and the fibrotic process [14]. Treatment with HMW-HA can also inhibit inflammatory response in several disease models, such as in HMW-HA attenuated inflammation and lung injury in a sepsis model of ventilated rats [79], as well as ozone-induced AHR [54, 55, 80, 81]. Based on recent data, HMW-HA is emerging as a viable therapeutic option in inflammatory airway disease, due to its anti-inflammatory and antiproliferative properties. Lingering concerns however will have to be addressed, such as the potential of degradation of HMW-HA into LMW-HA and thus exacerbation of the inflammatory process. It is worth noting that inhaled HMW-HA has been used for several years in Europe, with a remarkably good safety profile [82–85].

Certain environmental exposures and disease states have been shown to lead to breaking-down of HMW-HA into fragments (LMW-HA) that can stimulate expression of proinflammatory cytokines, chemokines, and growth factors [86] and increase AHR [54]. Increased levels of LMW-HA have been found in many lung disorders including asthma, pulmonary fibrosis, COPD, allergic alveolitis, interstitial lung disease, sarcoidosis, and pulmonary hypertension (reviewed in [87]) and another article in this edition (Lauer et al.), as well as other inflammatory diseases like rheumatoid arthritis [88]. LMW-HA can also induce angiogenesis and tumor progression [50, 89]. In aggregate, the effect of LMW-HA in tissue injury seems to be proinflammatory and rather deleterious, and attempts to block LMW-HA signaling in disease may constitute novel treatments.

Interestingly, oHA of different sizes (4–16 mers) have been shown to both stimulate and inhibit inflammatory responses depending on cell type and disease model. HA oligosaccharides have been shown to increase angiogenesis during wound healing [67], stimulate proinflammatory effects in synovial fibroblasts [66], and promote cell adhesion [51]. Conversely, there are many studies that show oHA to have beneficial effects such as reducing poly(I:C)/TLR3-induced inflammation [90], modulating the onset and course of inflammatory demyelinating disease by interfering with lymphocyte-vascular endothelial cell slow rolling [91], inhibiting HA-CD44 activation of kinases, and causing disassembly of large signaling complexes [92, 93], as well as retarding the growth of several tumor types and sensitizing resistant cancer cells to various drugs [50].

Finally, it is worth mentioning that, unlike some experimental conditions, cells and tissues in situ are exposed to a variety of HA sizes at once. It is possible that the effects of exposure to a HA size mix may be more than the sum of its separate size effects, as has been demonstrated experimentally (de la Motte C, personal communication).

## 8. Conclusion

Much is still unknown about the biological effects of HA in tissue homeostasis and the response to injury, and crucial insights into these effects will be gained by understanding the size dependence of HA signaling. Mechanistically, we need to understand how HA size may affect receptor clustering and affinity to receptors (especially receptor variant forms); whether there is a relation between HA size and the localization of HA (intra- versus extracellular, as well as cellular compartments); how simultaneous engagement of different HA sizes may modulate signaling; and the role of intracellular HA in cell behavior. Undoubtedly, we can look forward to exciting developments in the field of HA biology which will help fuel translational applications and medical advances.
